# RNA Sequencing of Tumor-Educated Platelets Reveals a Three-Gene Diagnostic Signature in Esophageal Squamous Cell Carcinoma

**DOI:** 10.3389/fonc.2022.824354

**Published:** 2022-05-09

**Authors:** Tiejun Liu, Xin Wang, Wei Guo, Fei Shao, Zitong Li, Yang Zhou, Zhihong Zhao, Liyan Xue, Xiaoli Feng, Yin Li, Fengwei Tan, Kai Zhang, Qi Xue, Shugeng Gao, Yibo Gao, Jie He

**Affiliations:** ^1^ Department of Thoracic Surgery, National Cancer Center/National Clinical Research Center for Cancer/Cancer Hospital, Chinese Academy of Medical Sciences and Peking Union Medical College, Beijing, China; ^2^ Cancer Institute of the Affiliated Hospital of Qingdao University, Qingdao Cancer Institute, Qingdao, China; ^3^ Department of Pathology, National Cancer Center/National Clinical Research Center for Cancer/Cancer Hospital, Chinese Academy of Medical Sciences and Peking Union Medical College, Beijing, China; ^4^ Department of Medical Examination for Cancer Prevention, National Cancer Center/National Clinical Research Center for Cancer/Cancer Hospital, Chinese Academy of Medical Sciences and Peking Union Medical College, Beijing, China; ^5^ State Key Laboratory of Molecular Oncology, National Cancer Center/National Clinical Research Center for Cancer/Cancer Hospital, Chinese Academy of Medical Sciences and Peking Union Medical College, Beijing, China

**Keywords:** esophageal squamous cell carcinoma, tumor-educated platelet, RNA sequencing, support vector machine, diagnosis

## Abstract

There is no cost-effective, accurate, and non-invasive method for the detection of esophageal squamous cell carcinoma (ESCC) in clinical practice. We aimed to investigate the diagnostic potential of tumor-educated platelets in ESCC. In this study, seventy-one ESCC patients and eighty healthy individuals were enrolled and divided into a training cohort (23 patients and 27 healthy individuals) and a validation cohort (48 patients and 53 healthy individuals). Next-generation RNA sequencing was performed on platelets isolated from peripheral blood of all participants, and a support vector machine/leave-one-out cross validation (SVM/LOOCV) approach was used for binary classification. A diagnostic signature composed of *ARID1A, GTF2H2*, and *PRKRIR* discriminated ESCC patients from healthy individuals with 91.3% sensitivity and 85.2% specificity in the training cohort and 87.5% sensitivity and 81.1% specificity in the validation cohort. The AUC was 0.924 (95% CI, 0.845–0.956) and 0.893 (95% CI, 0.821–0.966), respectively, in the training cohort and validation cohort. This 3-gene platelet RNA signature could effectively discriminate ESCC from healthy control. Our data highlighted the potential of tumor-educated platelets for the noninvasive diagnosis of ESCC. Moreover, we found that keratin and collagen protein families and ECM-related pathways might be involved in tumor progression and metastasis of ESCC, which might provide insights to understand ESCC pathobiology and advance novel therapeutics.

## Introduction

Esophageal cancer is one of the deadliest cancers worldwide, with a 5-year survival rate of 15%–25% (1–3). In China, esophageal squamous cell carcinoma (ESCC) accounts for more than 80% of all esophageal cancer cases (4). Currently, the most common modality to screen ESCC is endoscopy, which is invasive and inconvenient. Traditional tumor markers, including carcinoembryonic antigen (CEA), squamous cell carcinoma antigen (SCC), and cytokeratin 19 fragment (CYFRA21-1), have low sensitivity and specificity. Esophageal cytology samples have a high specificity but disappointing sensitivity for detection. Liquid biopsies represent a potential revolution in cancer diagnostics as a minimally invasive and sensitive alternative (5). Currently, blood-based liquid biopsy focuses on the evaluation of biomarker types, including circulating tumor DNA or microRNA, circulating tumor cells, and extracellular vesicles (6).

More recently, platelets are discovered as another biomarker (7). Tumor cells have the ability to educate platelets through cell-free or micro-vesicle-wrapped RNAs, thus enabling its better survival and development. Therefore, the concept of tumor-educated platelet (TEP) has arisen (8), and TEPs are believed to play significant roles in cancer initiation, progression, and metastasis (9, 10). Compared with other biomarkers mentioned, TEPs may offer certain advantages over other blood-based biosources, including their abundance and easy isolation, high-quality RNA, and capacity to process RNA in response to external signals. To date, TEPs from patients with different tumor types, including lung, brain, and breast cancers, have been tested (11). In this study, we used peripheral blood platelets of pre-operative ESCC patients to investigate the diagnostic potential of platelets in ESCC.

## Materials and Methods

### Study Cohort Design

In the present study, 71 ESCC patients and 80 healthy individuals were included. All participants were divided into a training cohort (23 patients and 27 healthy individuals) for diagnostic model construction and validation cohort (48 patients and 53 healthy individuals) for evaluation. This study was approved by the medical ethics committee and informed consents from participants were obtained.

A total of 71 ESCC patients were recruited from patients who received radical esophagectomy in the Department of Thoracic Surgery, Cancer Hospital, Chinese Academy of Medical Sciences. The 23 patients in the training cohort were collected between December 2017 and June 2018, while the 48 patients in validation cohort were collected between August 2018 and May 2019. The ESCC patients’ eligibility criteria included the following: pathological diagnostic ESCC through a biopsy procedure or surgical resection; no antineoplastic therapy, radiotherapy, or chemotherapy before surgery; and no previous esophageal cancer or other cancer history. ESCC tumor stage was determined using the 7th American Joint Committee on Cancer staging system (12).

A total of 80 healthy individuals were included. Among them, 25 were apparently healthy individuals who have undergone cancer screening examinations at the Department of Cancer Prevention, Cancer Hospital, Chinese Academy of Medical Sciences. They and the patients were 1:3 age- and sex-matched. Among the 25 healthy individuals, 8 individuals in the training cohort were collected between December 2017 and June 2018, while the 17 individuals in the validation cohort were collected between August 2018 and May 2019. In order to get more generalized results, we also enrolled 55 healthy individuals from different clinical centers reported in previous research (**Supplementary Data 1**) (7). Among them, 19 were assigned to the training cohort and 36 were assigned to the validation cohort randomly. The healthy controls’ eligibility criteria were as follows: they had no examination findings or history that suggested either malignancy or benign tumors after routine examinations, including chest x-rays or LDCT, abdominal B ultrasonic analysis, and complete blood tests (blood routine examination, blood biochemical analysis, tumor marker analysis, and blood coagulation system analysis).

### Blood Collection and Platelet Isolation

Peripheral blood was extracted into a 5-ml ethylenediaminetetraacetic acid (EDTA)-coated vacutainer tube and stored in the refrigerator at 4°C. All samples were processed within 6 h of collection. Gradient centrifugation combined with immunological screening was performed to isolate high-purity platelets (13). Then, the platelet pellet was washed in buffer and Miltenyi Biotec magnetic activated cell sorting (MACS) separators were used for CD45^+^ leukocyte depletion. Platelet quality and purity were assessed by microscopic and quantitative polymerase chain reaction (qPCR) determination of the CD45 (PTRPC) level (**Supplementary Figure S1**; **Supplementary Data 2**).

### RNA Extraction, Library Construction, and RNA Sequencing

Total RNA was extracted from isolated platelets frozen in 5 ml of PBS using Trizol Reagent and Qiagen RNeasy minElute spin column as described in the manufacturer’s instructions. The integrity of the total RNA was determined using an Agilent 2100 Bioanalyzer and RNA was quantified using a Thermo Scientific NanoDrop. Samples with RNA integrity number (RIN) above 7 were used for subsequent experiments. Details of library construction and RNA sequencing were listed in supplementary files (**Supplementary Datas 3, 4**).

RNA purification, reverse transcription, library construction, and sequencing were performed following the Illumina manufacturer’s instructions. The transcriptome coding regions captured from total RNA were prepared using the TruSeq RNA Exome Library Preparation Kit. Approximately 10 ng of high-quality RNA from fresh/frozen samples was used as input total RNA. Then, the RNA was fragmented into small pieces using divalent cations under elevated temperature. cDNA was generated from the cleaved RNA fragments using random priming during first- and second-strand synthesis and sequencing adapters were ligated to the resulting double-stranded cDNA fragments. The transcriptome coding regions were then captured from the library using sequence-specific probes to create the final library. After library construction, the Qubit 2.0 fluorometer dsDNA HS Assay (Thermo Fisher Scientific) was used to quantify concentration of the sequencing libraries, while the size distribution was analyzed using an Agilent Bioanalyzer 2100. Sequencing was performed using Illumina systems following Illumina-provided protocols for 2 × 150 paired-end sequencing.

### Quantitative RT-PCR

Quantitative RT-PCR was performed to validate the sequencing results. The reverse transcription reactions were performed using the TransScript All-in-One First-Strand cDNA Synthesis Supermix kit (Transgen Biotech Co., Ltd., Beijing, China) and PCR amplification was performed using the PerfectStart Green qPCR SuperMix kit (Transgen Biotech Co., Ltd., Beijing, China). Quantitative PCR reactions was performed on ABI 7900HT (Applied Biosystems, CA, USA) in a 10-μl reaction, in which reactions were activated at 94°C for 30 s, 45 cycles of 94°C for 5 s, 55°C for 15 s, and 72°C for 10 s. Primers used in this study are listed in **Table S1**.

### Data Statistics

FastQC (v0.11.2) was used to measure quality control. Skewer (v0.11.2) was used to eliminate adapter sequences for all samples. Clean reads above 75 bp were used for subsequent analysis. STAR (v2.3.0) software was used to map the filtered clean reads to the reference human genome (hg19) and generate BAM files. BAM files were input into RSEM software to generate original gene counts. All statistical analyses were performed in Rstudio (v1.2.1335), based on R (v3.6.1). Combat function in R-package sva was used to remove batch effect.

Remove unwanted variances (RUV) algorithm was used to adjust for systematic errors of unknown origin in high-dimensional data. The RUVg algorithm, one of the RUV algorithms, estimates the factors of unwanted variation using control genes and was used to avoid potential unwanted variations before performing differential expression analysis (14). The minimal redundancy and maximal relevance (MRMR) algorithm was used to select a feature subset that best characterized the statistical property of a classification variable (15). The support vector machine (SVM) algorithm and leave-one-out cross-validation (LOOCV) method were used to distinguish ESCC patient samples from controls (16).

Differences of variables followed a normal distribution between groups and were evaluated with the Student’s *t*-test. Logistic regression model was used to identify up- and downregulated genes between different groups. We conducted Pearson correlation analysis and calculated correlation coefficients using Pearson method between two continuous variables. If one of the variables is a continuous variable and the other is a discrete ordered variable, we conducted Spearman correlation analysis and calculated correlation coefficients using the Spearman method.

## Results

### Participant Characteristics, Platelet Quantity, and Platelet RNA Assessment

The clinical–pathological characteristics of participants are shown in **Table 1** (**Supplementary Data 5**). Only healthy controls from Cancer Hospital, Chinese Academy of Medical Sciences were statistically analyzed in **Table 1** in both training cohort and validation cohort. No significant differences in age and gender distribution were observed between the ESCC and control group (*p* > 0.05). Participants in the ESCC group tended to be former or current smokers compared to the control group in both cohorts (*p* < 0.001). There was no significant difference in distribution of tumor stage between the training and validation cohort.

**Table 1:** Characteristics of participants in the training cohort and validation cohort.

Variable		Training Cohort (*n* = 50)		Validation Cohort (*n* = 101)		*p*-value
		ESCC, *n* = 23 *n* (%)	Control, *n* = 27 *n* (%)	ESCC, *n* = 48 *n* (%)	Control, *n* = 53 *n* (%)	
Age (years) Mean ± SD		62.3 ± 8.6	58.1 ± 7.7	62.1 ± 8.67	59.2 ± 8.1	*p* > 0.05
Gender	Male (%)	20 (87.0%)	7 (87.5%)	43 (89.6%)	14 (82.4%)	
	Female (%)	3 (13.0%)	1 (12.5%)	5 (10.4%)	3 (17.6%)	
	*p*-value	*p* > 0.05				
Smoking Status						
	Non-smoker (%)	10 (43.5%)	5 (62.5%)	19 (39.6%)	10 (58.8%)	
	Current or former smoker (%)	13 (56.5%)	3 (37.5%)	29 (60.4%)	6 (35.3%)	
	Unknown (%)	0 (0%)	0 (0%)	0 (0%)	1 (5.9%)	
	*p*-value	*p* < 0.001				
Stage						
	I (%)	7 (30.4%)	–	14 (29.2%)	–	
	II (%)	6 (26.1%)	–	16 (33.3%)	–	
	III (%)	10 (43.5%)	–	18 (37.5%)	–	
	*p*-value	*p* > 0.05				

Only healthy controls from Cancer Hospital, Chinese Academy of Medical Sciences were statistically analyzed in **Table 1** in both the training cohort and validation cohort. ESCC: esophagus squamous cell carcinoma.

The median value of platelet counts was 216.0 × 10^9^/L in the ESCC group and 160.2 × 10^9^/L in the control group. Platelet counts in the ESCC group were significantly higher (*p* < 0.001, **Supplementary Figure S2A**). The average total platelet RNA isolated from 5 ml of peripheral blood was 288.5 ng (median values of 297.5 ng in the ESCC group and 254.2 ng in the control group). Total platelet RNA yield in the ESCC group was significantly higher than that in the control group (**Supplementary Figure S2B**, *p* < 0.001). There was no significant difference in RNA quality including both DV200 (percentage of RNA fragments that are >200 nucleotides in size) and OD_260_/OD_280_ between the ESCC and control groups (*p* > 0.05, **Supplementary Figures S2C, D**).

**Figure 1:**
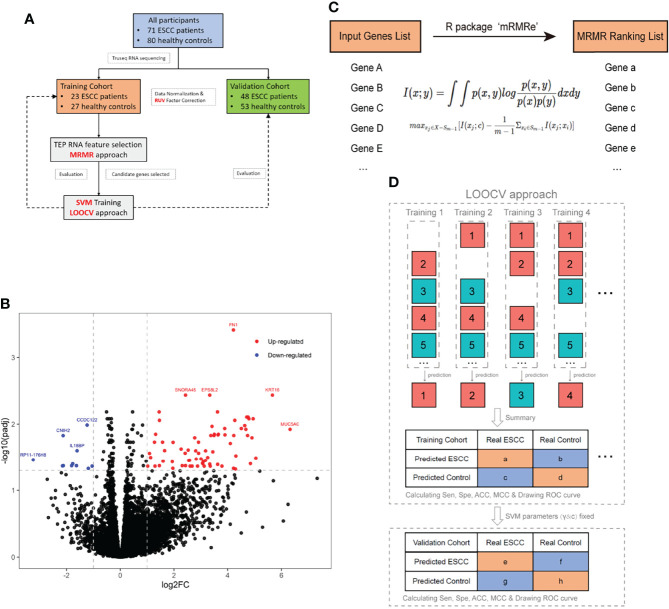
Schematic representation of analysis pipeline and core methods used in this study. **(A)** Flowchart of participant distribution and data processing procedure. **(B)** A logistic regression model was used to perform differential expression analysis between ESCC and the control group. Seventy-four red- and 11 blue-colored dots indicated upregulated genes and downregulated genes in ESCC, respectively (|fold change| >2 and *p*_adj_ < 0.05). *FN1, KRT16, EPS8L2, SNORA45*, and *MUC5AC* were most significantly upregulated and *CCDC122, CNIH2, IL18BP*, and *RP11-176H8* were most significantly downregulated. **(C)** Schematic representation of the MRMR approach. Top 200 differentially expressed genes input into R-package mRMRe to generate a gene-ranking list for features selection with maximum relevance and minimum redundancy. **(D)** Schematic representation of the SVM/LOOCV model training and validation. In the training cohort, the SVM algorithm was trained to get optimal parameters of gamma and cost by all samples minus one, while the remaining sample was used for blind classification until every sample has been predicted. The search range for gamma and cost parameters of SVM algorithm were 10^(-10:1) and 2^(1:10) respectively. After LOOCV approach was completed in the training cohort, gamma and cost parameters were fixed to do binary classification in the validation cohort. For both cohorts, we got a confusion matrix and ROC curve of diagnosis for ESCC. ACC, accuracy; ESCC, esophageal squamous cell carcinoma; LOOCV, leave-one-out cross-validation; MCC, Matthews correlation coefficient; MRMR, minimal redundancy and maximal relevance; SVM, support vector machine; ROC, receiver operative characteristics.

Platelet RNA sequencing yields a mean of 8,775 million bases and 58 million reads with an average 92% of Qphred scores above 30. Bioinformatic analysis followed a standard pipeline (**Figure 1A**). We excluded genes with less than five reads in more than 95% of sequenced samples, yielding a total of 16,629 genes for subsequent analysis (**Supplementary Figure S3A**). The correlation between library size and total gene counts was assessed. Two hundred and fourteen genes with a correlation coefficient greater than 0.7 in relation to library size were defined as negative control genes for RUV algorithm (**Supplementary Figure S3B**). Pearson correlation analysis between library size and total gene counts of those 214 control genes revealed a strong correlation (*r* = 0.9, *p* < 0.001) and no distribution difference was observed (**Supplementary Figure S3C**). The RUVg algorithm in the RUVseq R-package was used to avoid potential unwanted variations before performing differential expression analysis (**Supplementary Figure S3D**). A logistic regression model was used to perform differential expression analysis. Seventy-four upregulated and 11 downregulated RNAs (adjustive *p*-value < 0.05, |fold change| > 2, **Figure 1B**) were identified. It can be observed that upregulated RNAs tended to be more than downregulated RNAs in TEPs of ESCC compared with controls.

### Platelet RNA Signature for ESCC Diagnosis

The MRMR method was used to select the most effective rather than most significantly differentially expressed genes (DEGs). In this study, the top 200 DEGs by MRMR were input into R-package mRMRe to generate a gene-ranking list for features selection with maximum relevance and minimum redundancy (**Figure 1C**) (15).

We used the SVM algorithm and the LOOCV method to distinguish ESCC patient samples from controls using the top 200 genes. In this procedure, the SVM algorithm was trained by all samples in the training cohort (*n* = 50) minus one, while the remaining sample was used for blind classification. This procedure was repeated for *n* rounds until every sample has been predicted. By comparing the predicted class of samples with its actual class, sensitivity, specificity, accuracy (ACC), and Matthews correlation coefficient (MCC, frequently used in deep learning) were calculated to evaluate the classifier algorithm performance in the training cohort (**Figure 1D**). As the number of selected genes increased, ACC and MCC gradually increased and held in a stable level (**Supplementary Figures S4A, B**). Selecting the 3 top-ranked genes, namely, *ARID1A, GTF2H2*, and *PRKRIR*, produced an acceptable diagnostic performance with relatively small number of genes. The expression level of the three mRNAs measured by sequencing was verified by qRT-PCR (**Supplementary Figure S5** and **Supplementary Data 6**).

After selecting the optimal gene number, total samples in the training cohort were trained to obtain the optimal parameters of the SVM algorithm, whose search range for gamma and cost parameters were 10^(-10:1) and 2^(1:10), respectively. With fixed gamma and cost, we got an SVM prediction score for every sample in validation cohort.

**Figure 2:**
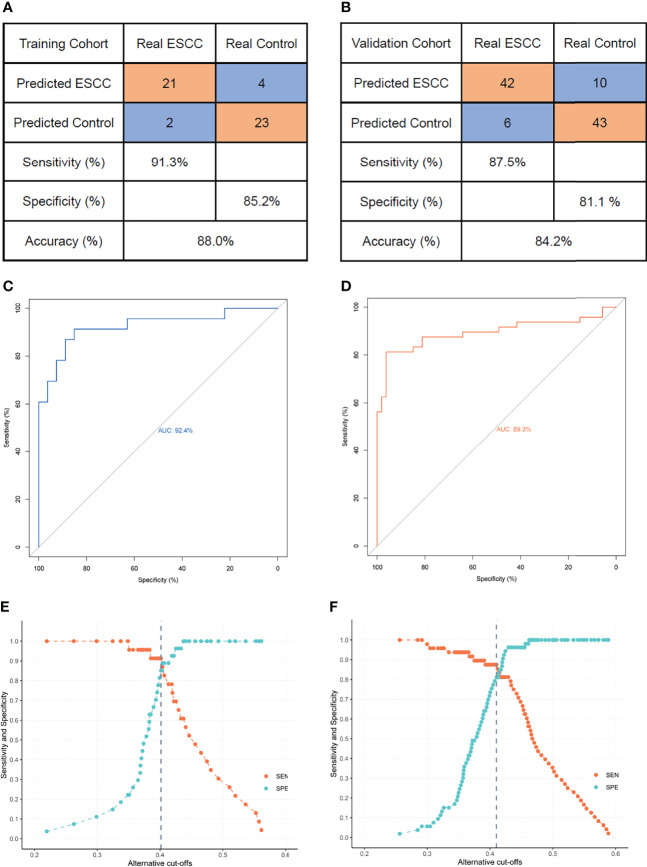
The three-gene panel diagnostic performance with alternative cutoffs in training cohort and validation cohort. **(A)** Confusion matrix of SVM/LOOCV diagnostic model between ESCC and the control group in the training cohort. The sensitivity, specificity, and ACC for the training cohort were 91.3%, 85.2%, and 88.0% respectively. **(B)** Confusion matrix of SVM/LOOCV diagnostic model between ESCC and the control group in validation cohort. The sensitivity, specificity, and ACC for the validation cohort were 87.5%, 81.1%, and 84.2%, respectively. **(C)** ROC curve of the SVM/LOOCV diagnostic model in the training cohort. Area under curve was 92.4% (95% CI, 0.84.5–0.956). **(D)** ROC curve of the SVM/LOOCV diagnostic model in validation cohort. Area under curve was 0.893% (95% CI, 0.821–0.966). **(E)** Sensitivity and specificity of the three-gene diagnostic model using alternative cutoffs in the training cohort. **(F)** Sensitivity and specificity of the three-gene diagnostic model using alternative cutoffs in validation cohort. Gray dashed line indicates the highest Youden index obtained when selecting the appropriate cutoff value. ACC, accuracy; AUC, area under curve; ESCC, esophageal squamous cell carcinoma; LOOCV, leave-one-out cross-validation; SVM, support vector machine; ROC, receiver operating characteristic.

Using receiver operating characteristic (ROC) curve, we obtained optimal threshold (or cutoff value) of prediction score to differentiate ESCC patients from healthy controls. Finally, for both cohorts, we got a confusion matrix and ROC curve of diagnosis for ESCC. The SVM model composed of *ARID1A, GTF2H2*, and *PRKRIR* yielded a sensitivity of 91.3% and a specificity of 85.2% for ESCC in the training cohort (**Figures 2A, E**) and a sensitivity of 87.5% and a specificity of 81.1% in the validation cohort (**Figures 2B, F**). The area under the curve (AUC) was 0.924 (95% CI, 0.845–0.956) and 0.893 (95% CI, 0.821–0.966) for the training and validation cohorts, respectively (**Figures 2C, D**).

### Cluster and Correlation Analysis Between Clinical Data and Diagnostic Genes

**Figure 3:**
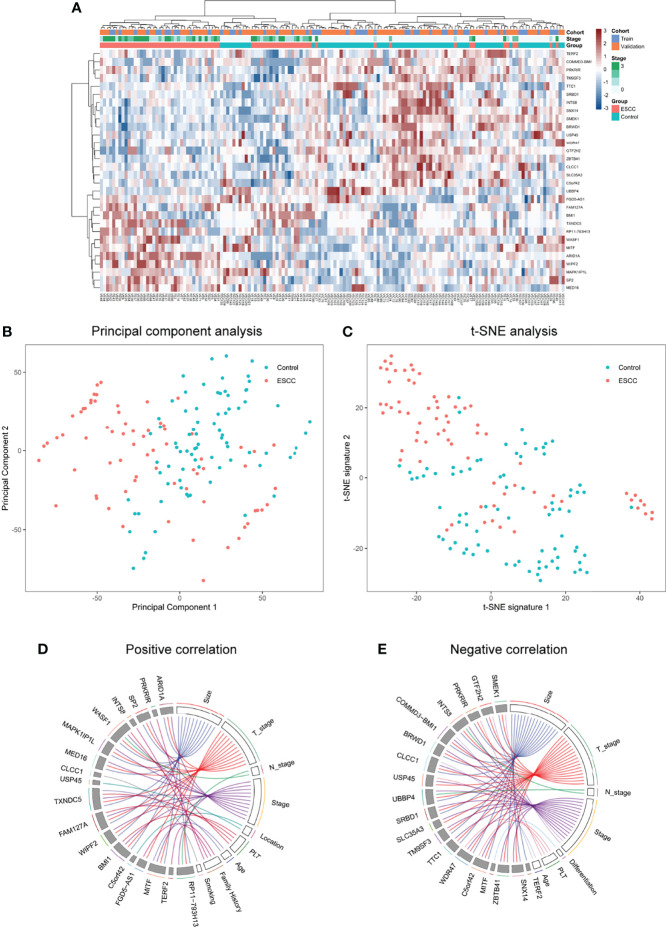
Top 30 gene signatures and clinicopathological relevance selected by MRMR approach. **(A)** Unsupervised hierarchical clustering of diagnostic top-ranked 30 gene signatures effectively discriminated between the ESCC group (*n* = 71) and the control group (*n* = 80). The 30-gene signature enables non-random clustering of all samples (*p* < 0.001, Fisher’s exact test). **(B)** Obviously different distributions of principal components analysis (PCA) between the control and ESCC group. Values of principal components were calculated using prcomp package in Rstudio. **(C)** Obviously different distributions of t-SNE visualization between the ESCC and control group. Values of t-SNE signature were calculated using Rtsne package in Rstudio. **(D)** Positive and **(E)** negative correlations between the diagnostic genes and clinical data. *p*-value above 0.05 was identified significantly correlated based on Pearson method between continuous data and Spearman method if one of the variables was not. Plots were drawn using the RCircos package in Rstudio. ESCC, esophageal squamous cell carcinoma; PCA, principal components analysis; t-SNE, t-distributed stochastic neighbor embedding.

The diagnostic curves in **Supplementary Figure S4** take an ascending trend when selecting the top ranking 30 genes. Therefore, we intended to explore the association between these 30 genes and clinical signature. We conducted cluster and correlation analysis between clinical data and differentially expressed top 30 genes selected by MRMR. Supervised clustering showed that this 30-gene signature effectively discriminates ESCC from control groups in both the training and validation cohort (*p* < 0.001) (**Figure 3A** and **Supplementary Table S2**). We also observed obviously different distributions of both principal components analysis (PCA) and t-distributed stochastic neighbor embedding (t-SNE) visualization between the control and ESCC group (**Figures 3B, C**). We also conducted correlation analysis between 30 genes and 11 clinical features, namely, tumor size, T stage, N stage, pathological stage, tumor location, differentiation, platelet counts, age, gender, family history, and smoking (pack years). Eighteen genes were positively correlated with 9 clinical features while 19 genes were negatively correlated with 7 clinical features. Most of the candidate genes selected for the diagnostic model showed a strong correlation with tumor size and stage. ARID1A tended to have a positive correlation with tumor size and tumor stage while GTF2H2 and PRKRIR tended to have a negative correlation with tumor size and tumor stage (**Figures 3D, E**).

### Functional Enrichment and Gene Co-Expression Network Analysis

**Figure 4:**
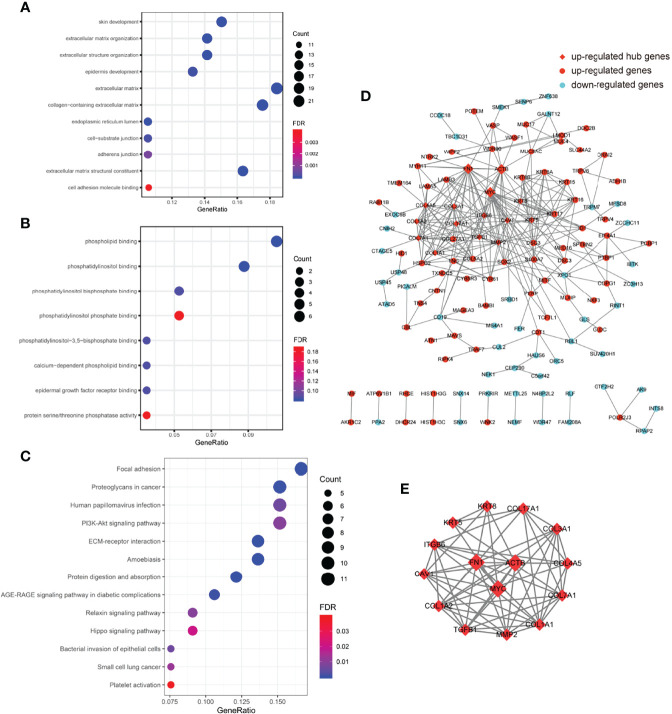
Functional enrichment and gene co-expression network analysis. **(A)** Top 12 significantly enriched GO terms (FDR < 0.001, count >10) ranked by gene ratio in BP (top 4 items), CC (middle 5 items), and MF (bottom 2 items) of 143 upregulated genes in platelets of the ESCC group compared to the control group. **(B)** Enriched GO terms (FDR < 0.2) of 80 downregulated genes in platelets of the ESCC group compared to the control group. All the enriched GO terms belonged to MF. **(C)** Enriched KEGG terms (FDR < 0.05) of 143 upregulated genes in platelets of the ESCC group compared to the control group. **(D)** Two hundred and twenty-three DEGs imported to String to generate a gene co-expression network containing 132 nodes and 281 wedges. Red-colored dots indicated 85 upregulated genes and blue-colored dots indicated 47 downregulated genes. Red square indicated hub genes (*FN1*, *ACTB*, and *MYC*) with highest values of degree. **(E)** Top 15 hub genes ranked by degree (>12) in Cystoscope were highly clustered and centered among *FN1*, *ACTB*, and *MYC*. BP, biological process; CC, cellular component; DEGs, differentially expressed genes; ESCC, esophageal squamous cell carcinoma; FDR, false discovery rate; GO, Gene Ontology; KEGG, Kyoto Encyclopedia of Genes and Genomes; MF, molecular function.

DEGs selected by the MRMR algorithm were not suitable for conducting GO, KEGG enrichment, and gene co-expression network analyses because this approach would exclude the so-called redundant genes that play pivotal roles in protein regulating pathways. Therefore, we use the logistic regression method to select DEGs with a *p*-value below 0.001 (adjust *p*-value below 0.075) and get 223 genes (144 upregulated and 77 downregulated) for subsequent analyses. Enriched GO terms, subdivided into an upregulated group (**Figure 4A**, **Supplementary Table S3**) and a downregulated group (**Figure 4B** and **Supplementary Table S4**), in biological process (BP), cellular components (CC), and molecular function (MF) were analyzed. Significantly enriched KEGG terms of upregulated genes are listed in **Figure 4C** (**Supplementary Table S5**) and no KEGG terms enriched of downregulated genes were found.

We also conducted gene co-expression network analysis (**Figure 4D**). We imported those 223 DEGs into String and exported a tsv file containing the node and interaction scores. Subsequently, the tsv file was imported into cytoscape to adjust nodes and wedges. Mcode and Cytohubba in cytoscape were used to identify hub genes based on the node degree. Eighty-six upregulated and 46 downregulated DEGs were involved in the network, which contained a major cluster and several gene pairs. Fifteen hub genes with a node degree above 12 were identified: *FN1*, *MYC*, *ACTB*, *COL1A1*, *COL7A1*, *KRT5*, *MMP2*, *COL1A2*, *ITGB6*, *COL17A1*, *COL4A5*, *COL3A1*, *TGFB1*, *CAV1*, and *KRT8* (**Figure 4E**). Notably, the top 15 hub genes showed a similar molecular background, and were mostly involved in extracellular matrix (ECM)-related pathways.

## Discussion

In this study, we found that platelet counts and platelet RNA yield in the ESCC group were significantly higher than those in the control group. Platelets are the most abundant component of peripheral blood and contain rich mRNAs, micro-RNAs, and noncoding RNAs (17, 18). Most RNA transcripts in platelets are derived from megakaryocytes. However, platelets can also ingest RNA molecules during circulation and/or interaction with other cell types. Once they enter the blood circulation from the primary location, cancer cells depend on platelets to protect them from shear forces and the assault of NK cells (19). Adhering with cancer cells, platelets secrete various chemokines, recruit myeloid cells, and arrest the tumor cells at the vascular wall to benefit cancer angiogenesis. In brief, platelets provide a permissive microenvironment for cancer cells to move and locate to a secondary tumor focus (20).

We constructed a 3-gene platelet RNA signature that could effectively distinguish ESCC from healthy controls. In previous literature, circulating microRNAs and methylated DNA markers (MDMs) were reported to have the potential for accurate detection of ESCC. The overall sensitivity and specificity of circulating microRNAs for detecting ESCC were 79.9% and 81.3%, respectively (21). In a pilot study involving 85 cases (76 esophageal adenocarcinoma and 9 ESCC), a 5-marker panel assayed from plasma detected 74% of esophageal cancer overall (74% of esophageal adenocarcinoma and 78% of ESCC) at a specificity of 91%. The diagnostic performance (Youden index of 0.765 in the training cohort and 0.686 in the validation cohort, respectively) of our study was higher than previous biomarkers (21, 22).

The three genes selected for our diagnostic panel, composed of *ARID1A*, *GTF2H2*, and *PRKRIR*, have different functions. *ARID1A* is the subunit gene of switching defective/sucrose non-fermenting protein complexes, which regulate gene activity by chromatin remodeling. PRKRIR is a regulator of interferon-induced serine/threonine protein kinase R (PKR), which may block the PKR-inhibitory function of DNAJC3, resulting in kinase activity restoration and suppression of cell growth. GTF2H2 is a core component of the general transcription and DNA repair factor TFIIH complex. *ARID1A* has been reported to be frequently mutated in a number of cancer types (23) and *PRKRIR* has been reported as a cancer-associated somatic mutation gene in ESCC (24). *GTF2H2* was reported to be associated with chemoresistance in non-small cell lung cancer (25) and breast cancer (26). However, the three genes have rarely been reported in TEPs. Li et al. (27) reported the evidence of TEP linc-GTF2H2-1 as a promising biomarker for lung cancer diagnosis, while *ARID1A* and *PRKRIR* have not been reported in TEPs in previous studies. No comprehensive literature has demonstrated the related functional mechanisms of these genes in TEPs. Platelets can interact with cancer cells and be educated *via* transfer of tumor-associated biomolecules. In the process of tumor-educated platelets, we presumed that several pathways were involved, including a direct connection between tumor cells and platelets, extracellular vesicle-dependent horizontal transmission from tumor cells to platelets, as well as megakaryocytes influenced by tumor cells (28).

The top 15 hub genes selected by network degree were mostly involved in ECM-related pathways. *FN1*, involved in cell adhesion and migration processes, has been reported to be crucial in tumor progression and as a potential biomarker in multiple cancers including colorectal cancer (29), gastric cancer (30, 31), ovarian cancer (32), and prostate cancer (33). *MYC*, a well-known oncogene with broad effects involved in cell cycle and tumor metabolism (34), has been reported as a prognostic biomarker in ESCC (35) and some other cancers including breast cancer (36) and lung cancer (37, 38). Beta-actin (*ACTB*), coding an abundant and highly conserved cytoskeleton structural protein and traditionally regarded as an endogenous housekeeping gene, is upregulated in esophageal cancer (39) and a variety of other cancers (40–45). Other highly clustered genes including collagen and keratin family protein coding genes, whose proteins were the main components of the ECM, were also upregulated in several cancer types (46, 47). As a crucial component of the tumor microenvironment, the ECM provides the mechanical support for the tissue, mediates cell–microenvironment interactions, and plays key roles in cancer cell invasion (48, 49).

However, our study has some limitations. Firstly, despite considering population differences and enrolling a cohort with multi-center healthy controls, most of the ESCC patients and a part of healthy controls are from Northern China. This is still a single-center study and needs further validation in multiple centers and a larger population. Secondly, participants in the ESCC group tended to be former or current smokers compared to the control group, and smoking seems to be a confounding factor that affects TEPs. Thirdly, since two-thirds of the enrolled patients had stage II/III ESCC, the early diagnostic value of these markers needs to be further evaluated in future studies. Moreover, the underlying mechanisms of the three genes selected for our diagnostic panel in predicting ESCC need to be further studied.

In conclusion, our study revealed a three-gene diagnostic signature in ESCC through RNA sequencing of TEPs. We provided the first insights into the potential of TEPs for the noninvasive diagnosis of ESCC. Moreover, our results potentially pave the way toward non-invasive and accurate methods for ESCC screening.

## Data Availability Statement

The original contributions presented in the study are publicly available. These data can be found here: NCBI, GSE197514.

## Ethics Statement

The studies involving human participants were reviewed and approved by National Cancer Center/Cancer Hospital, Chinese Academy of Medical Sciences and Peking Union Medical College National GCP Center for Anticancer Drugs, The Independent Ethics Committee. The patients/participants provided their written informed consent to participate in this study.

## Author Contributions

YG and JH jointly oversaw, coordinated, and provided funding for this study. YG conceptualized and designed experiments and data analysis. JH established the patient cohort. JH, XW, SG, QX, FT, YL, and KZ participated in collection and biobanking of specimen. LX and XF performed pathological review of specimen and assessment of IHC stain. TL, XW, WG, FS, ZL, YZ, and ZZ conducted extraction and quality control of platelet RNA. TL and XW performed data analysis, with support from YG. TL and YG participated in conceptual design and generation of plots and tables. The manuscript was written by TL and XW, edited by YG, and approved by all authors. All authors contributed to the article and approved the submitted version.

## Funding

This work was supported by the National Key R&D Program of China (2021YFC2501900 to YG), the National Natural Science Foundation of China (82122053 to YG, 82188102 to JH, 81972316 to YG), CAMS Initiative for Innovative Medicine (2021-I2M-1-067 to YG), Non-profit Central Research Institute Fund of Chinese Academy of Medical Sciences (2021-RC310-020 to YG) and Key-Area Research and Development Program of Guangdong Province (2021B0101420005 to YG).

## Conflict of Interest

The authors declare that the research was conducted in the absence of any commercial or financial relationships that could be construed as a potential conflict of interest.

The reviewer YS declared a shared affiliation with the authors to the handling editor at the time of the review.

## Publisher’s Note

All claims expressed in this article are solely those of the authors and do not necessarily represent those of their affiliated organizations, or those of the publisher, the editors and the reviewers. Any product that may be evaluated in this article, or claim that may be made by its manufacturer, is not guaranteed or endorsed by the publisher.

## Abbreviations

ACC, accuracy; AFP, alpha fetoprotein; AJCC, American Joint Committee on Cancer; AUC, area under the curve (AUC); CEA, carcinoembryonic antigen; CYFRA21-1, cytokeratin 19 fragment; ECM, extracellular matrix; EDTA, ethylenediaminetetraacetic acid; ESCC, esophageal squamous cell carcinoma; FDR, false discovery rate; GLM, general linear model; GO, Gene ontology; KEGG, Kyoto Encyclopedia of Genes and Genomes; LOOCV, leave-one-out cross validation; MACS, magnetic activated cell sorting; MCC, Matthews correlation coefficient; MCLC, Mid-1-related chloride channel; MRMR, minimal redundancy and maximal relevance; MISS, MAP kinase–interacting and spindle-stabilizing protein; PCA, principal components analysis; PRC1, polycomb group complex 1; PSA, prostate-specific antigen; RIN, RNA integrity number; ROC, receiver operating characteristic; RUV, remove unwanted variances; SCC, squamous cell carcinoma antigen; qPCR, quantitative polymerase chain reaction; SVM, support vector machine; TACS, tumor-associated collagen signatures; TEP, tumor-educated platelet; t-SNE, t-distributed stochastic neighbor embedding.
