# Supplementary material for: RNA Sequencing of Tumor-Educated Platelets Reveals a Three-Gene Diagnostic Signature in Esophageal Squamous Cell Carcinoma
Source: Front Oncol. 2022 May 9;12:824354. doi: 10.3389/fonc.2022.824354 (PMC9124963; doi:10.3389/fonc.2022.824354)
Supplement: Supplementary file 1 [file DataSheet_1.doc]

**Supplementary Tables**

Table S1 Primers used in this study

| Primers | Accession number | Sequence (5′→3′) |
| --- | --- | --- |
| GAPDH forward | NM_002046.7 | AAATCCCATCACCATCTTCC |
| GAPDH reverse |  | ATGACCCTTTTGGCTCCC |
| ARID1A forward | NM_006015.6 | GAACGGGGAGAAGACCCT |
| ARID1A reverse |  | GGAACTGCTGGTTGACTGG |
| GTF2H2 forward | NM_001515.3 | ATGAAAGCCATTACAAAGAGTT |
| GTF2H2 reverse |  | ATCCTGGTCAGATAAAGAAGC |
| PRKRIR forward | NM_004705.4 | GGGGCAAACCTCTGATGTCTT |
| PRKRIR reverse |  | TTGTGGCTTCCTCAAACCAAA |

Table S2 Differentially expressed top 30 genes selected for SVM/LOOCV diagnostic model for ESCC

| Gene name | Ensembl ID |
| --- | --- |
| ARID1A | ENSG00000117713 |
| GTF2H2 | ENSG00000145736 |
| PRKRIR | ENSG00000137492 |
| SMEK1 | ENSG00000100796 |
| SP2 | ENSG00000167182 |
| INTS8 | ENSG00000164941 |
| WASF1 | ENSG00000112290 |
| MAPK1IP1L | ENSG00000168175 |
| MED16 | ENSG00000175221 |
| COMMD3-BMI1 | ENSG00000269897 |
| BRWD1 | ENSG00000185658 |
| CLCC1 | ENSG00000121940 |
| USP45 | ENSG00000123552 |
| UBBP4 | ENSG00000263563 |
| TXNDC5 | ENSG00000239264 |
| SRBD1 | ENSG00000068784 |
| FAM127A | ENSG00000134590 |
| WIPF2 | ENSG00000171475 |
| SLC35A3 | ENSG00000117620 |
| TM9SF3 | ENSG00000077147 |
| TTC1 | ENSG00000113312 |
| WDR47 | ENSG00000085433 |
| BMI1 | ENSG00000168283 |
| C5orf42 | ENSG00000197603 |
| FGD5-AS1 | ENSG00000225733 |
| MITF | ENSG00000187098 |
| ZBTB41 | ENSG00000177888 |
| SNX14 | ENSG00000135317 |
| TERF2 | ENSG00000132604 |
| RP11-793H13 | ENSG00000267281 |

Abreactions: ESCC: esophageal squamous cell carcinoma; LOOCV: leave-one-out cross validation; SVM: support vector machine.

Table S3 Enriched GO terms of 143 up-regulated genes in platelets of ESCC

| **ID** | **Description** | **Ontology** | **GeneRatio** | **FDR** | **Genes ID** |
| --- | --- | --- | --- | --- | --- |
| GO:0043588 | skin development | BP | 17/113 | 1.02E-06 | COL1A1/PERP/DHCR24/KRT17/LAMA5/DSG3/DSC3/S100A7/COL1A2/COL3A1/KRT8/KRT15/KRT6B/KRT5/KRT16/COL5A2/KRT6A |
| GO:0030198 | extracellular matrix organization | BP | 16/113 | 7.74E-07 | TNC/MMP2/TGFB1/COL1A1/COL7A1/ITGB6/FN1/LAMA5/MYH11/HSPG2/CCN1/COL1A2/COL3A1/COL4A5/LAMB3/COL5A2 |
| GO:0043062 | extracellular structure organization | BP | 16/113 | 1.63E-06 | TNC/MMP2/TGFB1/COL1A1/COL7A1/ITGB6/FN1/LAMA5/MYH11/HSPG2/CCN1/COL1A2/COL3A1/COL4A5/LAMB3/COL5A2 |
| GO:0008544 | epidermis development | BP | 15/113 | 7.69E-05 | COL17A1/PERP/COL7A1/KRT17/LAMA5/DSG3/DSC3/S100A7/KRT8/KRT15/KRT6B/KRT5/KRT16/LAMB3/KRT6A |
| GO:0031012 | extracellular matrix | CC | 21/114 | 8.99E-11 | TNC/COL17A1/MMP2/TGFB1/COL1A1/COL7A1/FN1/LAMA5/HSPG2/CCN1/S100A7/MUC4/AZGP1/COL1A2/COL3A1/MUC17/COL4A5/COL27A1/LAMB3/MUC2/COL5A2 |
| GO:0062023 | collagen-containing extracellular matrix | CC | 20/114 | 8.78E-11 | TNC/COL17A1/MMP2/TGFB1/COL1A1/COL7A1/FN1/LAMA5/HSPG2/CCN1/S100A7/AZGP1/COL1A2/COL3A1/MUC17/COL4A5/COL27A1/LAMB3/MUC2/COL5A2 |
| GO:0005788 | endoplasmic reticulum lumen | CC | 12/114 | 1.26E-05 | TNC/COL17A1/COL1A1/COL7A1/FN1/CCN1/COL1A2/COL3A1/COL4A5/COL27A1/COL5A2/TXNDC5 |
| GO:0030055 | cell-substrate junction | CC | 12/114 | 1.59E-04 | TNC/COL17A1/ACTB/CAV1/CBL/TRPV4/WASF1/ITGB6/VASP/TNS4/HSPG2/S100A7 |
| GO:0005912 | adherens junction | CC | 12/114 | 8.58E-04 | BAIAP2L1/TNC/ACTB/CAV1/CBL/TRPV4/WASF1/ITGB6/VASP/TNS4/HSPG2/S100A7 |
| GO:0005201 | extracellular matrix structural constituent | MF | 17/104 | 2.15E-14 | TNC/COL17A1/COL1A1/COL7A1/FN1/LAMA5/HSPG2/CCN1/MUC4/COL1A2/COL3A1/MUC17/COL4A5/COL27A1/LAMB3/COL5A2/MUC5AC |
| GO:0050839 | cell adhesion molecule binding | MF | 11/104 | 4.22E-3 | BAIAP2L1/CBL/FN1/VASP/LAMA5/COL3A1/PCBP1/NLGN2/SPTBN2/EPS8L2/RAB11B |

Abreactions: ESCC: esophageal squamous cell carcinoma; FDR: false discovery rate; GO: Gene ontology.

Table S4 Enriched GO terms of 80 down-regulated genes in platelets of ESCC

| **ID** | **Description** | **Ontology** | **GeneRatio** | **FDR** | **Genes ID** |
| --- | --- | --- | --- | --- | --- |
| GO:0005543 | phospholipid binding | MF | 6/57 | 0.08 | COL1A1/PERP/DHCR24/KRT17/LAMA5/DSG3/DSC3/S100A7/COL1A2/COL3A1/KRT8/KRT15/KRT6B/KRT5/KRT16/COL5A2/KRT6A |
| GO:0035091 | phosphatidylinositol binding | MF | 5/57 | 0.08 | TNC/MMP2/TGFB1/COL1A1/COL7A1/ITGB6/FN1/LAMA5/MYH11/HSPG2/CCN1/COL1A2/COL3A1/COL4A5/LAMB3/COL5A2 |
| GO:1902936 | phosphatidylinositol bisphosphate binding | MF | 3/57 | 0.09 | TNC/MMP2/TGFB1/COL1A1/COL7A1/ITGB6/FN1/LAMA5/MYH11/HSPG2/CCN1/COL1A2/COL3A1/COL4A5/LAMB3/COL5A2 |
| GO:1901981 | phosphatidylinositol phosphate binding | MF | 3/57 | 0.19 | COL17A1/PERP/COL7A1/KRT17/LAMA5/DSG3/DSC3/S100A7/KRT8/KRT15/KRT6B/KRT5/KRT16/LAMB3/KRT6A |
| GO:0080025 | phosphatidylinositol-3,5-bisphosphate binding | MF | 2/57 | 0.09 | TNC/COL17A1/MMP2/TGFB1/COL1A1/COL7A1/FN1/LAMA5/HSPG2/CCN1/S100A7/MUC4/AZGP1/COL1A2/COL3A1/MUC17/COL4A5/COL27A1/LAMB3/MUC2/COL5A2 |
| GO:0005544 | calcium-dependent phospholipid binding | MF | 2/57 | 0.09 | TNC/COL17A1/MMP2/TGFB1/COL1A1/COL7A1/FN1/LAMA5/HSPG2/CCN1/S100A7/AZGP1/COL1A2/COL3A1/MUC17/COL4A5/COL27A1/LAMB3/MUC2/COL5A2 |
| GO:0005154 | epidermal growth factor receptor binding | MF | 2/57 | 0.09 | TNC/COL17A1/COL1A1/COL7A1/FN1/CCN1/COL1A2/COL3A1/COL4A5/COL27A1/COL5A2/TXNDC5 |
| GO:0004722 | protein serine/threonine phosphatase activity | MF | 2/57 | 0.19 | TNC/COL17A1/ACTB/CAV1/CBL/TRPV4/WASF1/ITGB6/VASP/TNS4/HSPG2/S100A7 |

Abreactions: ESCC: esophageal squamous cell carcinoma; FDR: false discovery rate; GO: Gene ontology.

Table S5 Enriched KEGG terms of 143 up-regulated genes in platelets of ESCC

| **ID** | **Description** | **GeneRatio** | | | **FDR** | | **Genes ID** | |
| --- | --- | --- | --- | --- | --- | --- | --- | --- |
| hsa04510 | Focal adhesion | | 11/66 | 2.25E-05 | | 3371/60/857/1277/3694/2335/7408/3911/1278/1287/3914 | |  |
| hsa05205 | Proteoglycans in cancer | | 10/66 | 1.38E-04 | | 60/4313/7040/857/1277/867/2335/4609/3339/1278 | |  |
| hsa05165 | Human papillomavirus infection | | 10/66 | 5.12E-03 | | 3371/1277/3694/2335/525/3911/83439/1278/1287/3914 | |  |
| hsa04151 | PI3K-Akt signaling pathway | | 10/66 | 7.91E-03 | | 3371/1277/3694/2335/3911/4609/4915/1278/1287/3914 | |  |
| hsa04512 | ECM-receptor interaction | | 9/66 | 4.21E-06 | | 3371/1277/3694/2335/3911/3339/1278/1287/3914 | |  |
| hsa05146 | Amoebiasis | | 9/66 | 7.71E-06 | | 7040/1277/2335/3911/1278/1281/1287/3914/4583 | |  |
| hsa04974 | Protein digestion and absorption | | 8/66 | 2.93E-05 | | 1308/1277/1294/1278/1281/1287/85301/1290 | |  |
| hsa04933 | AGE-RAGE signaling pathway in diabetic complications | | 7/66 | 3.36E-04 | | 4313/7040/1277/2335/1278/1281/1287 | |  |
| hsa04926 | Relaxin signaling pathway | | 6/66 | 7.91E-03 | | 4313/7040/1277/1278/1281/1287 | |  |
| hsa04390 | Hippo signaling pathway | | 6/66 | 1.71E-02 | | 60/7040/3397/4609/83439/6657 | |  |
| hsa05100 | Bacterial invasion of epithelial cells | | 5/66 | 5.12E-03 | | 60/857/867/8936/2335 | |  |
| hsa05222 | Small cell lung cancer | | 5/66 | 1.04E-02 | | 2335/3911/4609/1287/3914 | |  |
| hsa04611 | Platelet activation | | 5/66 | 3.28E-02 | | 60/1277/7408/1278/1281 | |  |

Abreactions: ESCC: esophageal squamous cell carcinoma; FDR: false discovery rate; KEGG: Kyoto Encyclopedia of Genes and Genomes.
