# Supplementary material for: RNA Sequencing of Tumor-Educated Platelets Reveals a Three-Gene Diagnostic Signature in Esophageal Squamous Cell Carcinoma
Source: Front Oncol. 2022 May 9;12:824354. doi: 10.3389/fonc.2022.824354 (PMC9124963; doi:10.3389/fonc.2022.824354)
Supplement: Supplementary file 2 [file DataSheet_2.doc]

**Supplementary Figures**


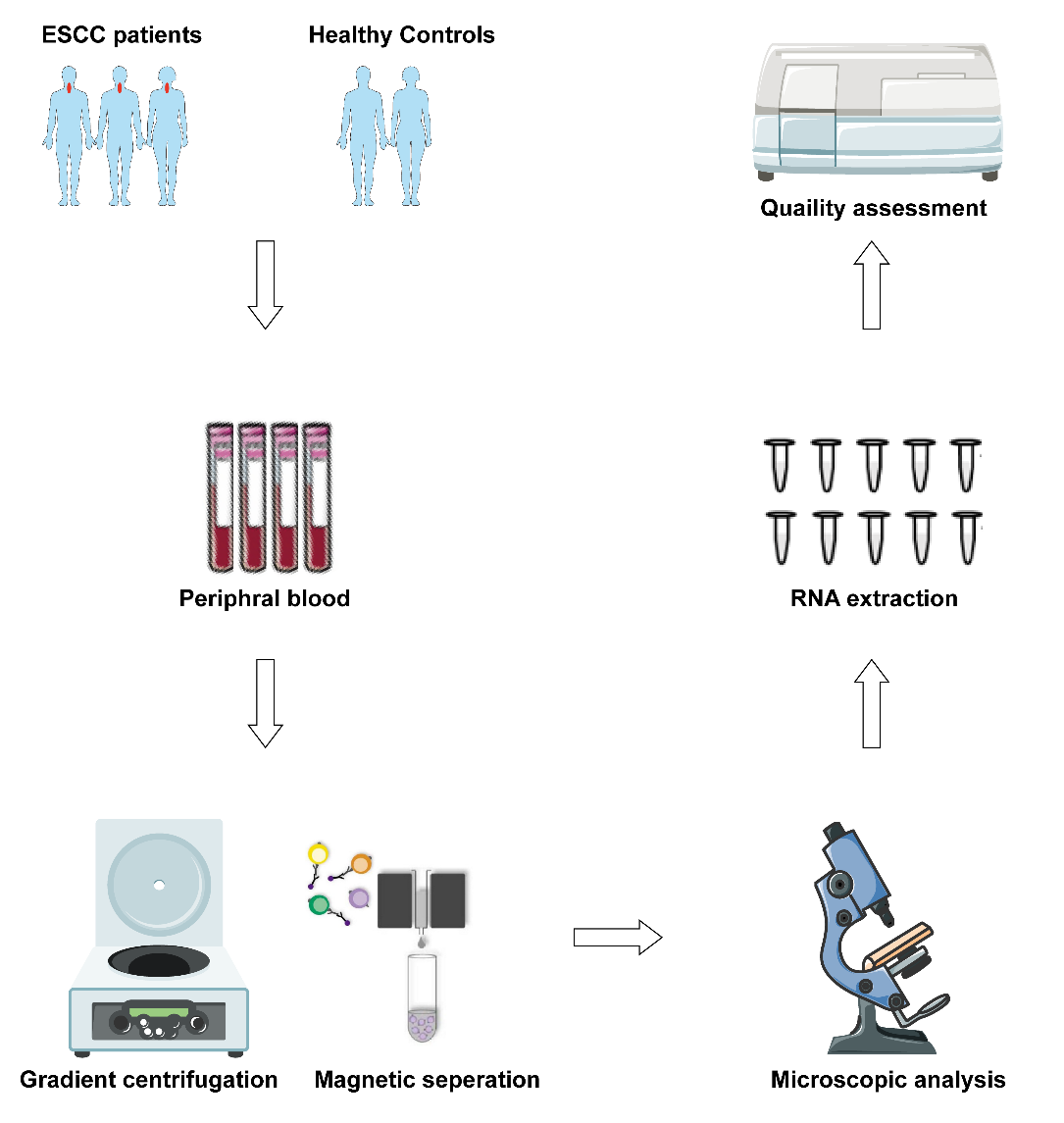


**Figure.S1 Schematic representation of blood sample possessing and RNA extraction.**

Blood samples were collected from both ESCC patients and healthy controls. Gradient centrifugation and magnetic separation were combined to isolate high-purity platelets. Platelet quality and purity were assessed by microscopic and reverse transcription polymerase chain reaction (RT-PCR) determination of the CD45 (PTRPC) level. The integrity of the total RNA was determined using an Agilent 2100 Bioanalyser and RNA was quantified using a Thermo Scientific NanoDrop.

Abbreviations: ESCC: ESCC: esophageal squamous cell carcinoma; RT-PCR: reverse transcription polymerase chain reaction (RT-PCR).


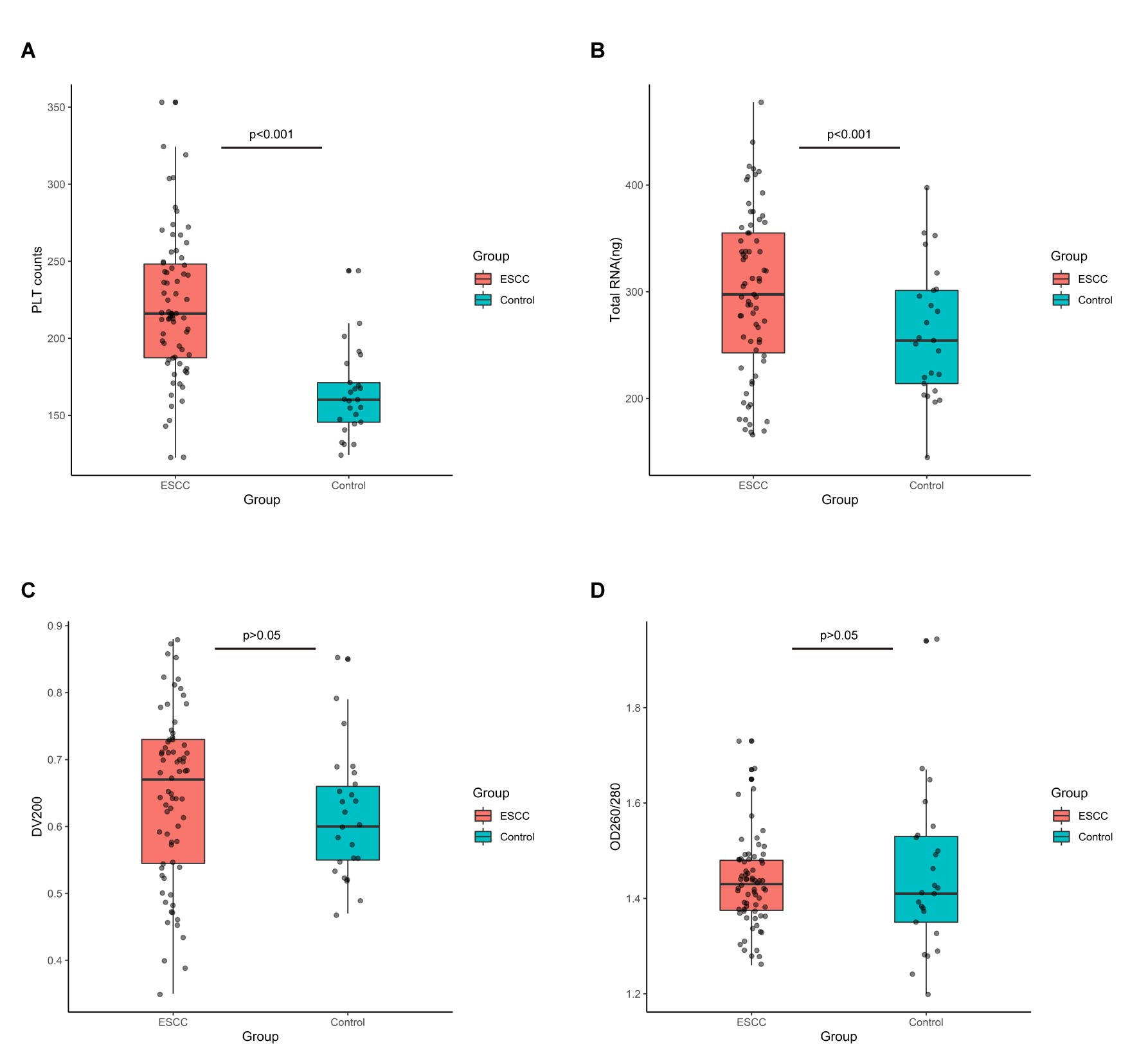


**Fig. S2 Quantity and quality assessment of peripheral blood platelets and extracted RNAs.**

**(A)** Platelet counts of peripheral blood in ESCC group were significantly higher than control group (*P*<0.001, Student’s t test). **(B)** Total platelet RNA yield in ESCC group was significantly higher than control group (*P*<0.001, Student’s t test). **(C)** There was no significant difference in DV200 (percentage of RNA fragments that are >200 nucleotides in size) of platelet RNA between ESCC group and control group (*P*>0.05, Student’s t test). **(D)** There was no significant difference in OD260/OD280 of platelet RNA between ESCC group and control group (*P*>0.05, Student’s t test). Abbreviations: ESCC: esophageal squamous cell carcinoma; OD: optical density.


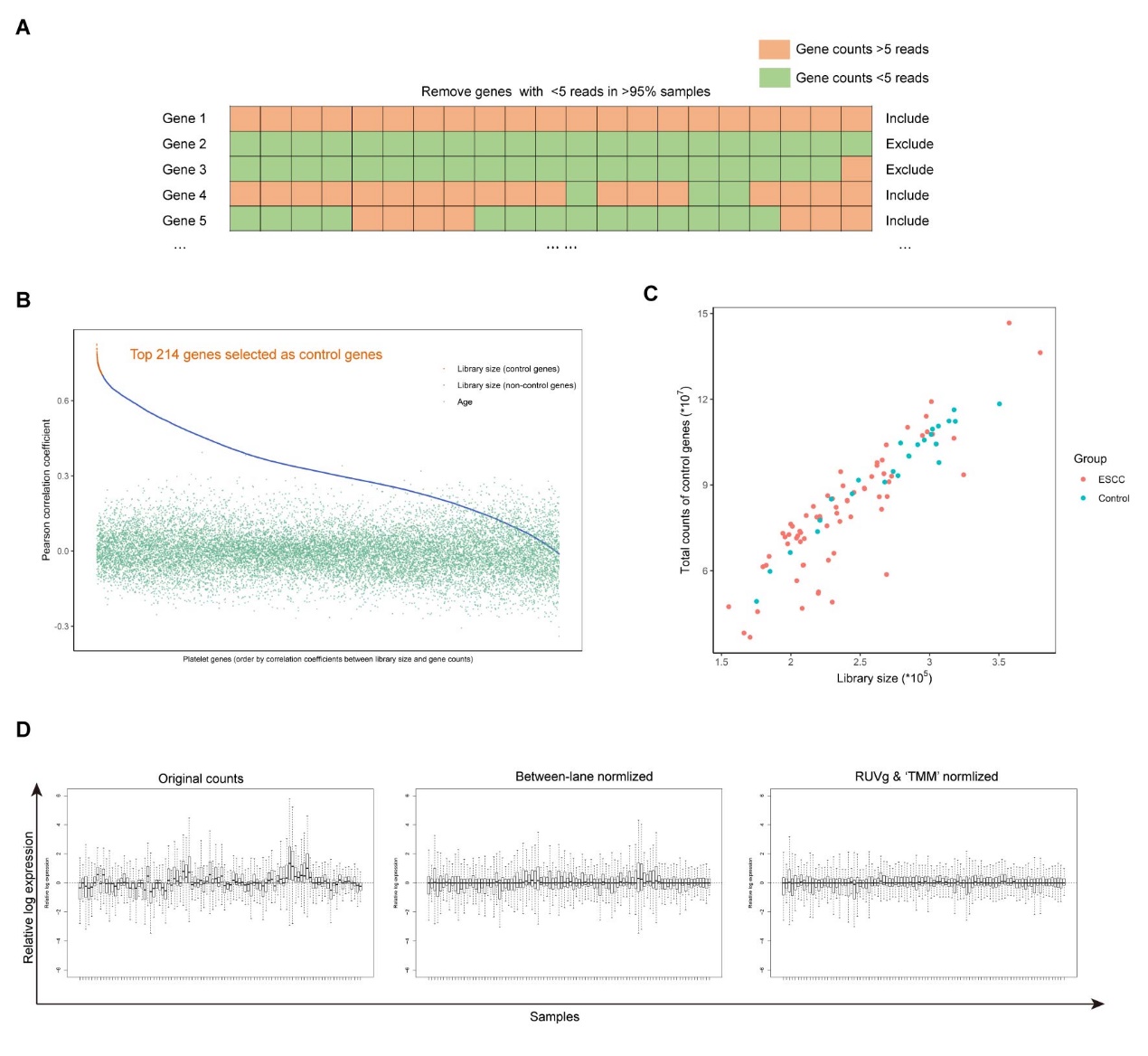


**Fig. S3 Bioinformatic normalization procedure of original sequencing data.**

**(A)** Genes with less than five reads in more than 95% of sequenced samples were excluded, yielding a total of 16629 genes for subsequent analysis. (**B)** Pearson correlation coefficient between age and gene counts distributed mostly between -0.3 to 0.3, thus unsuitable as control factors. Pearson correlation coefficient between library size, age and gene counts in descending order, top 214 (yellow line, correlation coefficient value> 0.7) genes were selected as control genes. (**C)** Library size and total gene counts of 214 control genes were strong-correlated (Pearson correlation coefficient=0.90, p=2.2e-16). No significant distribution was observed between ESCC and control group. (**D)** The boxplots of relative log expression of original gene counts showed clear variances in both median, upper-quartile and lower-quartile (left). Normalized by betweenLaneNormalization function of R-package EDASeq, variances of median, upper-quartile and lower-quartile in most but a small number of samples have been eliminated (middle). Using RUVg function of R-package RUVSeq and 214 control genes selected in supplementary fig. 2b, unwanted variances across all samples have been removed (right). Abbreviations: ESCC: esophageal squamous cell carcinoma; RUV: remove unwanted variances.


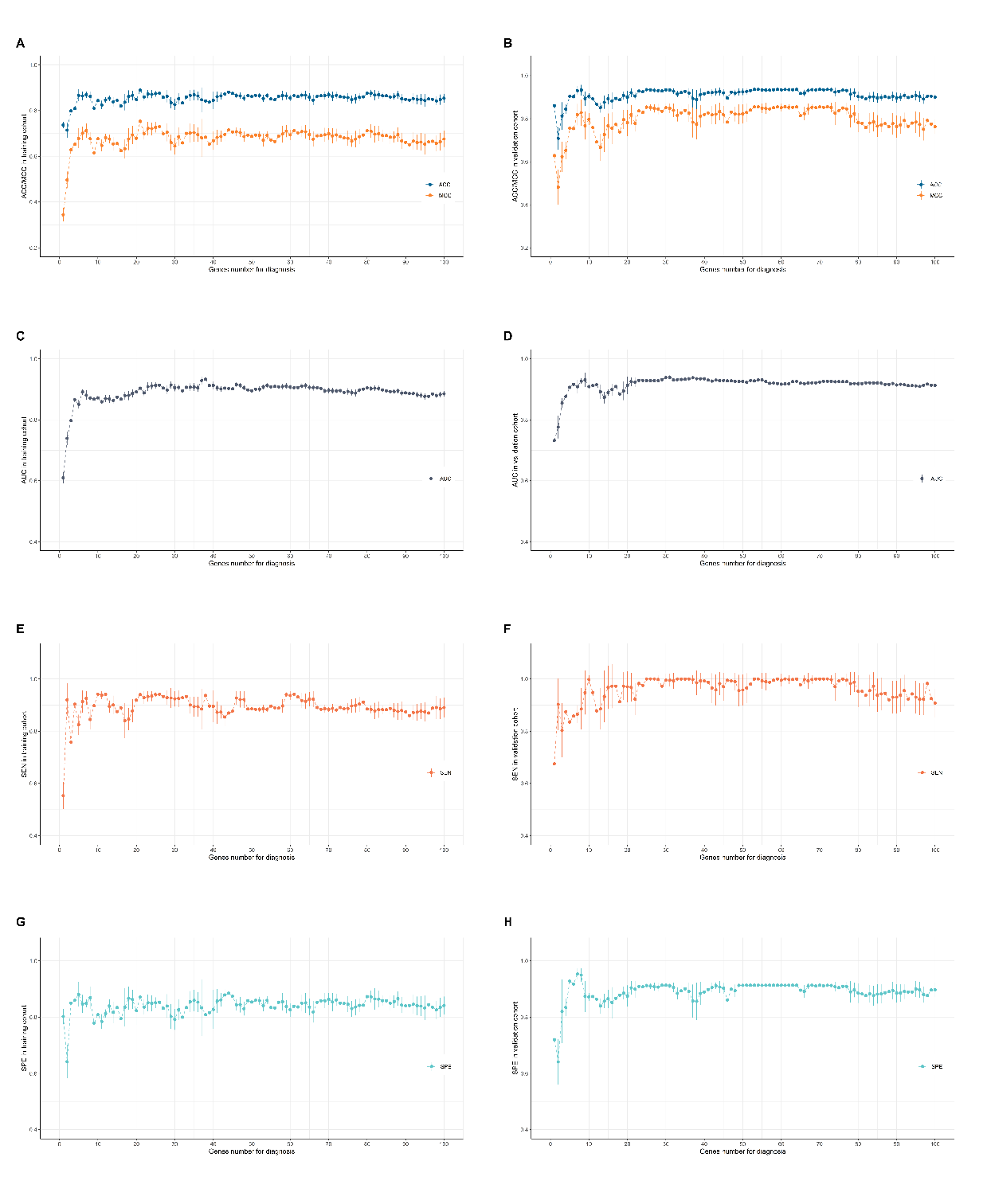


**Fig. S4 Diagnostic performance of SVM/LOOCV algorithm in training cohort and validation cohort.** MRMR-approach selected genes were used for ESCC diagnostic model training to select optimal genes number. As the number of selected genes increased, ACC and MCC gradually increased and held in a stable level. Selecting the top-ranked 3 genes produced an acceptable ACC and MCC values, with as few as possible of genes. We calculated ACC, MCC, AUC, SEN and SPE in both training cohort (A, C, E, G) and validation cohort (B, D, F, H). As a random procedure may exist in SVM process, all indexes were calculated 100 times for each selected gene number and presented with an error line. Abbreviations: ACC: accuracy; AUC: area under curve; ESCC: esophageal squamous cell carcinoma; LOOCV: leave-one-out cross validation; MCC: Matthews correlation coefficient; MRMR: minimal redundancy and maximal relevance; SEN: sensitivity; SPE: specificity; SVM: support vector machine.


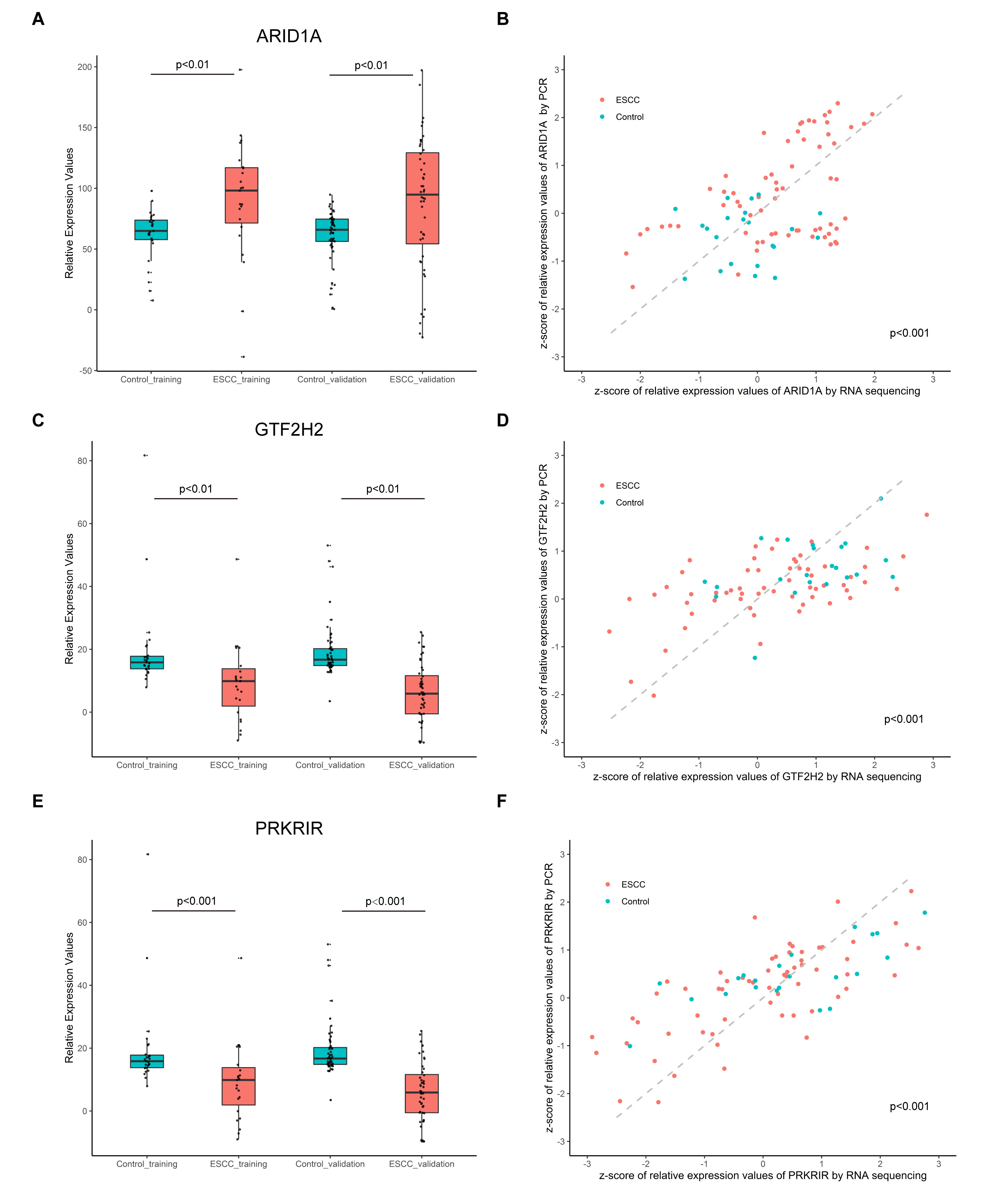


**Fig. S5 Significantly different distribution of selected mRNAs between ESCC and control groups in both training cohort and validation cohort.** Expression values of *ARID1A*(A), *GTF2H2*(C), *PRKRIR*(E) in control and ESCC group. P values were calculated by Student’s test. RNA sequencing results of *ARID1A*(B), *GTF2H2*(D), *PRKRIR*(F) were validated by qRT-PCR. GAPDH was used as spike-in for normalization. Both sequencing expression values and PCR △CT values were transformed to z-score. For all mRNAs, z-score values by sequencing method and PCR method were significantly correlated (p<0.001, Pearson correlation test). Abbreviations: ESCC: esophageal squamous cell carcinoma; qPCR: quantitative polymerase chain reaction.
